# Supplementary material for: DNA sequencing of whole human cytomegalovirus genomes from formalin-fixed, paraffin-embedded tissues from congenital cytomegalovirus disease cases
Source: PLoS One. 2025 May 30;20(5):e0318897. doi: 10.1371/journal.pone.0318897 (PMC12124853; doi:10.1371/journal.pone.0318897)
Supplement: S1 Table — (DOCX) [file pone.0318897.s001.docx]

**S1 Table. Characteristics of extracts used to generate sequence datasets.**

| **Case no.** | **Sample age^a^** | **Dataset name^b^** | **DNA load (ng/µL)** | **A260/ A280** | **HCMV load^c^** | **Human load^d^** | **HCMV load/ human load** |
| --- | --- | --- | --- | --- | --- | --- | --- |
| **184** | 1 | 184P_fp | 17.8 | 1.41 | 11,911 | 672 | 17.72 |
|  |  | 184P_gr | 27.6 | 2.31 | 27,698 | 960 | 28.85 |
|  |  | 184R_fp | NA^e^ | 1.76 | 740 | 3,861 | 0.19 |
|  |  | 184R_gr | 81.9 | 1.94 | 435 | 1,073 | 0.41 |
| **70** | 2 | 70P_fp | 13.2 | 1.07 | 731 | 2,061 | 0.35 |
|  |  | 70P_gr | 25.3 | 1.33 | 117 | 781 | 0.15 |
|  |  | 70R_fp | 47.2 | 1.76 | 100,732 | 43,162 | 2.33 |
|  |  | 70R_gr | NA | 1.76 | 32,962 | 3,130 | 10.53 |
| **150** | 2 | 150P_fp | NA | NA | NA | NA | NA |
|  |  | 150P_gr | 28.7 | 2.32 | 17,726 | 939 | 18.88 |
| **413** | 2 | 413P_fp | 8.6 | 1.41 | 50,974 | 21,992 | 2.32 |
|  |  | 413P_gr | 177.6 | 1.69 | 6,302 | 785 | 8.03 |
|  |  | 413R_fp | 27.7 | 1.84 | 55,849 | 45,635 | 1.22 |
|  |  | 413R_gr | 28 | 2.22 | 6,773 | 1,116 | 6.07 |
| **35** | 5 | 35P_fp | 2.28 | 1.61 | 116,574 | 13,663 | 8.53 |
|  |  | 35P_gr | 68.3 | 2.16 | 12,693 | 525 | 24.18 |
|  |  | 35R_fp | 11.1 | 1.76 | 14,647 | 25,693 | 0.57 |
|  |  | 35R_gr | 37.9 | 2.05 | 745 | 562 | 1.33 |
| **239** | 5 | 239P_fp | 16.9 | 1.38 | 11,204 | 2,722 | 4.12 |
|  |  | 239P_gr | 25.4 | 2.45 | 5,601 | 694 | 8.07 |
|  |  | 239R_fp | 45.9 | 1.8 | 4,135 | 498 | 8.3 |
|  |  | 239R_gr | NA | NA | NA | NA | NA |
| **473** | 6 | 473P_fp | 0.629 | 1.38 | 3,822 | 1,119 | 3.42 |
|  |  | 473P_gr | 80.8 | 2.08 | 2,974 | 485 | 6.13 |
| **660** | 6 | 660R_fp | 16.6 | 1.6 | ND^f^ | ND | NA |
|  |  | 660R_gr | 36 | 2.22 | 86 | 141 | 0.61 |
| **68** | 7 | 68R_fp | 21.6 | 1.54 | 2,198 | 1,241 | 1.77 |
|  |  | 68R_gr | 49 | 2.11 | 2,730 | 685 | 3.99 |
| **124** | 7 | 124P_fp | NA | NA | NA | NA | NA |
|  |  | 124P_gr | 18.7 | 2.39 | 751 | 135 | 5.56 |
|  |  | 124R_fp | NA | NA | NA | NA | NA |
|  |  | 124R_gr | 92.1 | 2.03 | 125 | 125 | 1 |

^a^ FFPE sample age (years) from collection to sequencing.

^b^ The case no. is suffixed by P (placenta) or R (kidney) and then by _fp (FormaPure extraction kit) or _gr (GeneRead extraction kit).

^c^ IU/µL of HCMV UL97.

^d^ Copies/µL of human *FOXP2*.

^e^ Not available.

^f^ Not detected.
