# Supplementary material for: DNA sequencing of whole human cytomegalovirus genomes from formalin-fixed, paraffin-embedded tissues from congenital cytomegalovirus disease cases
Source: PLoS One. 2025 May 30;20(5):e0318897. doi: 10.1371/journal.pone.0318897 (PMC12124853; doi:10.1371/journal.pone.0318897)
Supplement: S1 Fig — (DOCX) [file pone.0318897.s002.docx]

**
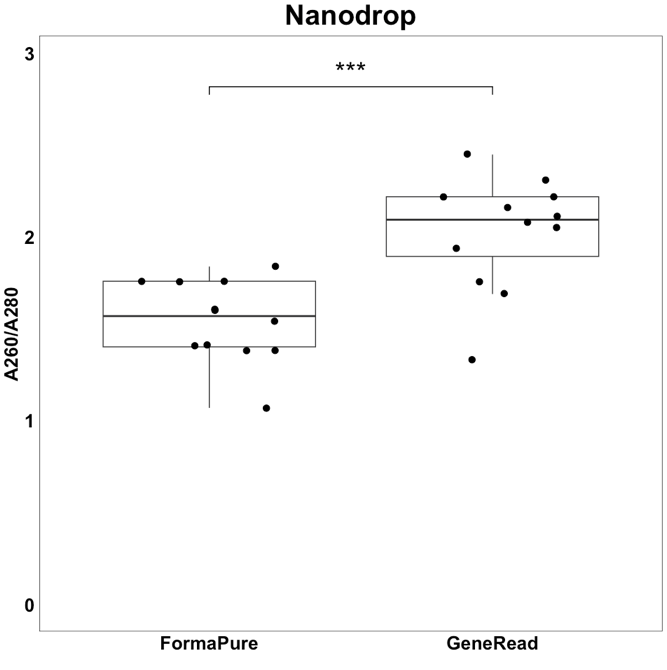

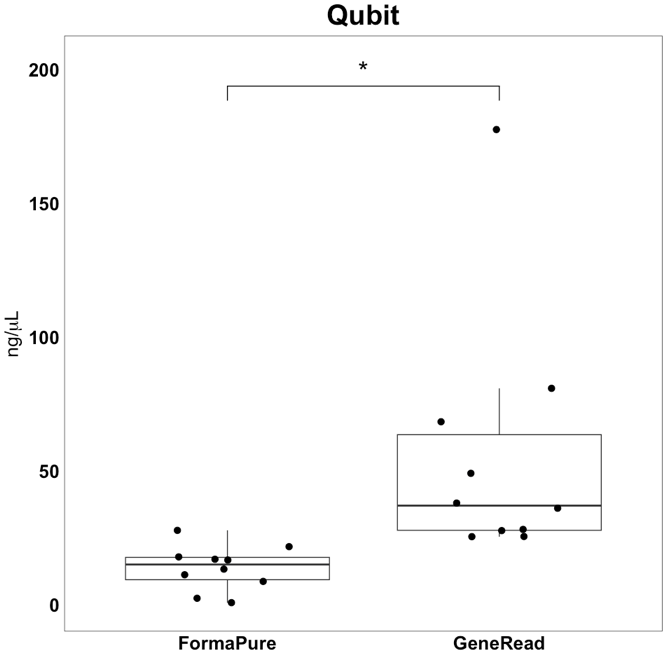
Figure S1. Plots characterising FFPE extracts prepared using the FormaPure or GeneRead kits and sequence data generated from these extracts.**

**(b)**

**(a)**

(a) DNA concentration in extracts measured by Qubit; (b) ratio of absorbance values at 260 nm and 280 nm (A260/A280) measured by Nanodrop; and (c) average coverage depth of the HCMV strain Merlin genome (GenBank accession AY446894.2) by sequence data generated from extracts obtained using both kits. In each plot, the thick black line represents the mean, the box represents the first to third quartiles, and the whiskers extend to the 5 % and 95 % quantiles (* p<0.05, *** p<0.001; ns, not significant; two-tailed Student’s t-test).


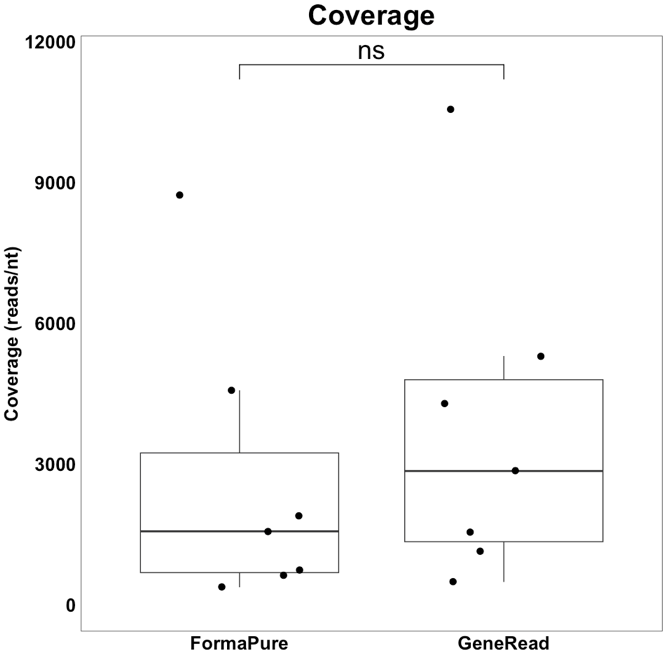


**(c)**
