# Supplementary material for: DNA sequencing of whole human cytomegalovirus genomes from formalin-fixed, paraffin-embedded tissues from congenital cytomegalovirus disease cases
Source: PLoS One. 2025 May 30;20(5):e0318897. doi: 10.1371/journal.pone.0318897 (PMC12124853; doi:10.1371/journal.pone.0318897)
Supplement: Attachment — Submitted filename: Main reviever comments.docx [file pone.0318897.s003.docx]

Review of **“DNA sequencing of whole human cytomegalovirus genomes from formalin-fixed, paraffin-embedded tissues from congenital cytomegalovirus disease cases”**

Over all I believe the study is solid, however, limited in scope.
There is no statistical analysis, which in this case I belive is fine.
I think the DNA quality data is necessary to included in one form or another and study would benefit with a comparison of DNA quality and usability of the sample for WGS of HCMV genome.
The revies is written in an intelligible fashion of standard english.

The authors aims to assess the usability of archival FFPE tissue samples for whole genome sequence of human cytomegalovirus genomes. And they claim that this is indeed very feasible.

- Though this is not a very ambitious aim, I believe the presented data shows that this is possible.
- To improve the study, I think it would be more interesting to investigate what make a FFPE samples usable for the WGS of HCMV.

Sixteen FFPE samples from placental or kidney tissue of ten cases collected in the period of 2008-2018 are tested.

Two DNA extraction kits were used.

- I would like the authors to explain further why these two methods were used and comments on any differences were identified in DNA quality or downstream results? Is any of the kits better for special tissue?

The Authors find The DNA to be of sufficient quality for all but one sample.

- I would like to know how this quality is assessed and what the requirements is to be of sufficient quality. Please provide the DNA quality of all samples, at least as supplementary.

Three datasets were not meeting the threshold requirements of genotyping.

- Are there anything special with these data sets/ samples? How was the DNA quality?

For figure 1. The The abbriviations …”P_fp” and “R_gr” should be explained as they are in table 2.

The authors claim in the discussion that “it is possible that low HCMV load, rather than poor quality DNA, was the main contributor to low read coverage in older samples.”

- I would as mentioned before like to see the DNA quality data, and maybe do a direct comparison of HCMV load, DNA quality and quality of output data.
- It could also be interesting to look into storage time of the samples?
